# Probing Broken Time-Reversal Symmetry in 2D Materials with Tailored-Light Photocurrent Generation

**DOI:** 10.1021/acsnano.5c17857

**Published:** 2026-03-17

**Authors:** Daniel M. B. Lesko, Tobias Weitz, Simon Wittigschlager, Selina Nöcker, Weizhe Li, Peter Hommelhoff, Ofer Neufeld

**Affiliations:** † Department of Physics, Friedrich-Alexander-Universität Erlangen-Nürnberg (FAU), Erlangen 91058, Germany; ‡ Fakultät für Physik, Ludwig-Maximilians-Universität, München 80539, Germany; § Technion Israel Institute of Technology, Faculty of Chemistry, Haifa 3200003, Israel

**Keywords:** time-reversal symmetry, magnetism, ultrafast spectroscopy, photocurrent generation

## Abstract

The bulk photogalvanic effect represents a powerful tool for generating photocurrents without external bias in light-matter systems that lack inversion symmetry. While these photocurrents are used in electronic applications such as current sources, switches, and photovoltaics, their presence can also be employed to probe material properties in and out of equilibrium. Here we advance this path of bulk photogalvanic photocurrent spectroscopy by utilizing tailored laser fields for ultrafast photocurrent generation to study time-reversal-symmetry (TRS) broken phases of matter in 2D systems. Combinations of bichromatic linearly polarized laser beams that separately respect mirror and time-reversal symmetries, individually precluding photocurrents, can break symmetries and generate photocurrents when combined. We show in graphene, both theoretically and experimentally, that specific choices of the relative polarization angle and two-color phase impose a forbidden photocurrent selection rule in TRS-invariant systems, as the tailored light maintains TRS while breaking all other symmetries. We then employ state-of-the-art ab initio simulations to validate this physical mechanism and, crucially, predict a broken photocurrent selection rule in materials with intrinsically broken TRS, such as the 2D magnet CrI_3_, creating a background-free signal for TRS-broken phenomena such as magnetism and Chern physics. Our work highlights a method for probing TRS-broken phases of matter in an ultrafast time-resolved manner, not requiring the application of external magnetic fields or even circularly polarized electric fields.

## Graphical abstract

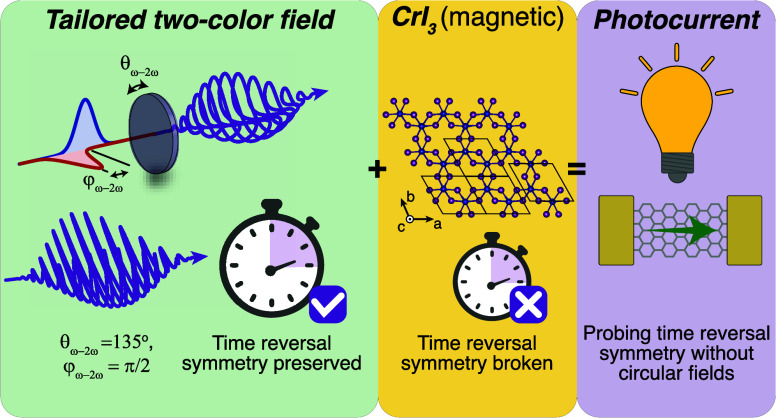

In recent years, ultrafast and nonlinear optics has become an incredibly useful tool for probing the unique properties of quantum materials both in and out of equilibrium. For example, high harmonic spectroscopy of solids^1–3^ has shed light on ultrafast exciton dynamics,^4–8^ superconductivity,^9^ strongly correlated materials,^10,11^ topology,^12–17^ and nonlinear phononics.^10,18,19^ Besides high harmonic spectroscopy, other nonlinear optical observables can also be employed to probe material properties, such as photocurrents generated from the bulk photogalvanic effect (BPGE).^20,21^ This effect allows for generating photocurrents without external bias in light-matter systems that lack inversion symmetry. Importantly, BPGE originating in inversion-symmetric materials must result from broken inversion and time-reversal symmetry of the applied fields. More generally, bulk photogalvanic photocurrents result from shift and/or injection currents, the former originating from a shift in the center of mass of the conduction band population (due to the inversion asymmetry of a material), while the latter is from asymmetric conduction band population throughout the Brillouin zone (and can occur in inversion-symmetric materials).

Bulk photogalvanic photocurrents can thus provide a sensitive probe to both the material composition and the optical field, resulting in a versatile tool for investigating light-matter interactions. Interestingly, BPGE currents were shown to be quantized in Weyl semimetals^22,23^ as well as give access to dynamical evolution of Floquet topological insulators (FTIs)^24,25^ and quantum materials,^26–29^ hinting toward their practical applicability.

One particularly interesting realization of nonlinear optical spectroscopies employs laser driving with tailored fields, which permits breaking or inducing symmetries beyond those intrinsic to the crystal lattice.^30^ Bichromatic optical fields represent a well-known and experimentally straightforward example and have extensively been used in both gas and solid-state high harmonic generation,^31–36^ provided enhanced control of steady-state Floquet phases,^37–40^ and access to suboptical-cycle changes to the band structure.^41–43^ Furthermore, bichromatic light can be used to generate and coherently control photocurrents in centrosymmetric solids,^44–50^ valley selectivity,^43,51,52^ ultrafast magnetic impulses,^53,54^ and in-plane magnetization.^55^ Similarly, two-frequency phonon modes have been proposed for manipulating crystal symmetries and orbital magnetism.^56^ However, so far, these methods have not been employed to probe ultrafast magnetism^57–59^ or magnetic phases of matter.

Most commonly, magnetic phenomena are measured by utilizing a probe that breaks time-reversal symmetry (TRS) itself, accomplished with external magnetic fields or monochromatic circularly polarized optical electric fields. These methods give rise to circular-dichroic responses, e.g., in transient absorption,^60,61^ perturbative nonlinear optics,^62^ or photoemission spectroscopies.^63,64^ While Faraday rotation spectroscopies generally use linearly polarized probes (that do not break TRS), they can be sensitive to interface effects as well as higher order nonlinearities and absorption,^65,66^ and can be challenging to use with atomically thin materials or with one-dimensional topological states. Investigating phases of matter with broken TRS with probes that do not break TRS remains a fundamental challenge, especially for ultrafast spectroscopies where probes might affect the dynamics being measured (e.g., generate ultrafast magnetism instead of probing existing magnetism).

Here, we develop and experimentally demonstrate a novel spectroscopic technique utilizing BPGE photocurrents driven by femtosecond-tailored light to probe time-reversal symmetry broken phases in 2D materials. Key to our analysis is the application of a bichromatic ω-2ω field, which allows altering the characteristic symmetries and chirality of the probe^40^ in a manner that is not possible with monochromatic fields. By tuning the relative angle and phase between two linearly polarized waves, the beam can either (i) break all point-group symmetries of the crystal (including TRS) and generate a BPGE current or (ii) break all point-group symmetries besides TRS, leading to a TRS-based photocurrent suppression selection rule. By measuring the angle- and phase-dependent photocurrent, we map the symmetry elements of the material phase in a precise and controllable manner. Importantly, the latter bichromatic field can probe TRS without inherently breaking it, starkly different from traditional ultrafast spectroscopies employing circular fields. We experimentally measure these selection rules for graphene and further validate this physical mechanism with ab initio time-dependent density functional theory (TDDFT) calculations and an analytical theory. We then employ ab initio simulations in the 2D magnet CrI_3_,^67^ showing that the photocurrent selection rule is lifted due to the broken TRS intrinsic to the material, leading to unique system-specific behavior. Finally, we find that this selection rule is broken in simulations of a Floquet topological insulator (FTI, a Chern insulator with broken TRS^68,69^ ) generated by irradiation of graphene with a circularly polarized dressing mid-infrared laser. Our work establishes BPGE photocurrents driven by bichromatic fields as a background-free probe for TRS-broken phases that can be used for ultrafast spectroscopy of Chern (quantized) physics and magnetism.

## Spectroscopic Concept

We begin by outlining the concept of the employed bichromatic ω – 2ω tailored-light (Figure 1a), with both carrier waves fixed to linear polarization states. This bichromatic field has two remaining key degrees of freedom: The relative polarization angle between the two beams (θ_ω–2ω_), and the relative phase of the two carrier components (φ_ω–2ω_). Crucially, we can tune θ_ω–2ω_ and φ_ω–2ω_ by adjusting the angle of a two-color half-wave plate and the thickness of a calcite plate, respectively, in a two-color inline interferometer (Figure 1b).^70^ This effectively alters the characteristic shape and Lissajous figure of the resulting field, tuning its symmetries (Figure 1c). These mirror and time symmetries are directly visible in the symmetry elements of the Lissajous plots of the electromagnetic field. Simultaneously, we measure the resulting photocurrents arising along the *x*-axis (Figure 1b). Further experimental details on the setup and bichromatic field synthesis are delegated to the Methods section.

**Figure 1:**
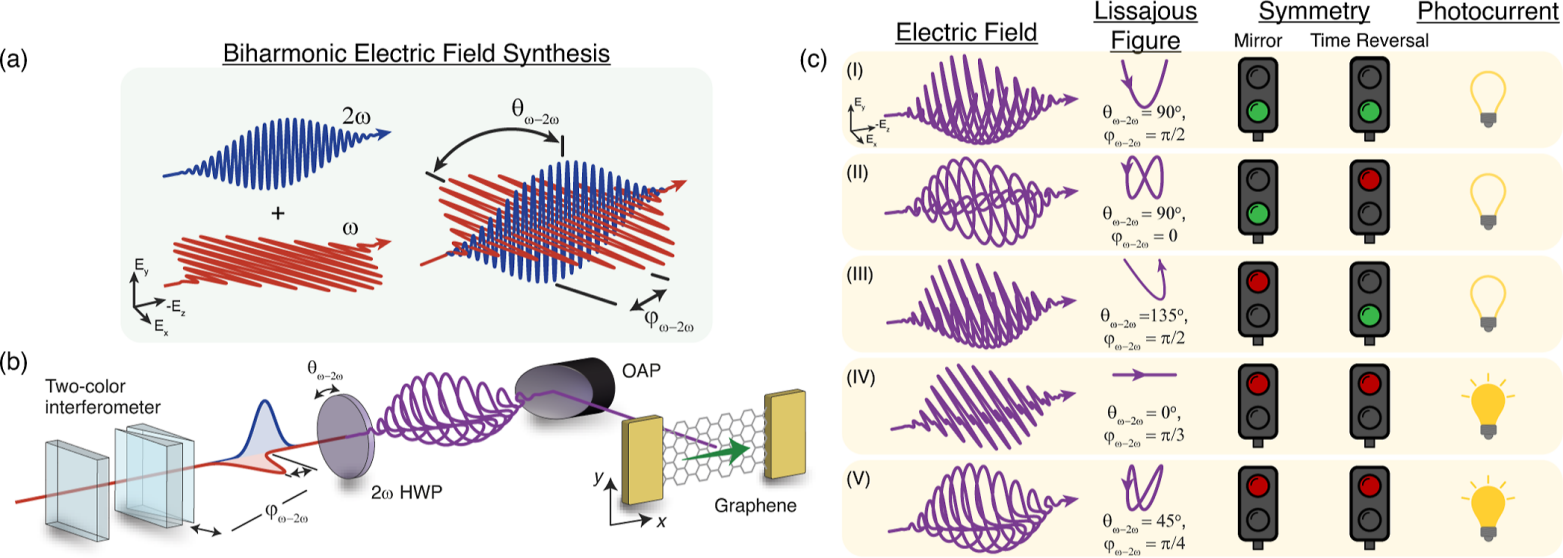
Tailored light two-color symmetry breaking. (a), Linearly polarized optical harmonics are combined with control over the relative angle (θ_ω–2ω_) and the two-color phase (φ_ω–2ω_). (b) Sketch of the experimental setup. The phase (φ_ω–2ω_) between the copropagating ω and 2ω pulses is controlled in a collinear two-color interferometer. With a dichroic half-waveplate (2ω HWP), we control the angle of the 2ω field with the ω polarization fixed (θ_ω–2ω_). Using an off-axis parabolic mirror (OAP), we focus the resulting waveform on a monolayer graphene strip, mounted in a vacuum chamber (not shown). We measure θ_ω–2ω_ and φ_ω–2ω_-dependent photocurrents via gold electrodes attached to the graphene strip. c, When combined, these fields can break mirror symmetry (in the *x* direction) and/or time-reversal symmetry, with broken symmetry indicated by the red traffic light, depending on both θ_ω–2ω_ and φ_ω–2ω_, generating a photocurrent in graphene as indicated by the yellow lightbulb. We showcase exemplary broken symmetry waveforms in cases I–V.

These degrees of freedom in the light field allow us to explore two main symmetry elements in the light-matter interaction: a mirror symmetry along the *y­(-z)*-plane (MS, labeled σ_*y*_, invoking *x* → –*x*) and a time-reversal symmetry (TRS, invoking **E**(*t*) → **E**(−*t*)). For instance, for θ_ω–2ω_ = 90° and φ_ω–2ω_ = π/2 (Figure 1c­(i)) the optical electric field exhibits a distinct dynamical mirror symmetry^71^ such that we have the symmetry relation **E**(*t*) = σ_*y*_·**E**(*t* + *T*/2), where σ_*y*_ is coupled to time-translations of half an optical period (*T* = 2π/ω). When this field interacts with graphene, the full light-matter Hamiltonian respects all symmetry elements because graphene itself is both mirror- and time-translation symmetric. This symmetry in the Hamiltonian leads to a photocurrent suppression along the *x*-direction (Figure 1c­(i)).

Importantly, the separate control of the angle and phase of the beam components allows for complete and independent MS and TRS breaking (Figure 1c­(ii-v)), which cannot be achieved solely with monochromatic optical fields. That is, by proper choice of the laser parameters, we can either invoke or break each of these symmetry elements separately, allowing us to probe the material symmetries without having the probe pulse break the symmetry being investigated. This allows utilizing the bichromatic drive to explore TRS breaking phenomena in a unique manner—without the presence of any other symmetry elements in the system such as other mirror or inversion elements. This is a crucial point in our analysis—by allowing selective degrees of freedom to be scanned in a multidimensional manner, we can develop a spectroscopy technique that is sensitive to independent symmetry-breaking elements, since by probing the system’s characteristic response as the field transitions in between the symmetric and symmetry-broken states. This principle is similar to symmetry-breaking spectroscopy techniques previously proposed for chiral phenomena,^72–75^ but here we utilize nonlinear photocurrent generation that removes the need for atomic-strength optical fields and is uniquely sensitive to TRS phenomena. Importantly, using photocurrent suppression to investigate symmetries should be less sensitive to material decoherence pathways (e.g., electron–electron and electron–phonon scattering) as the current signal accumulates and should be insensitive to phase-matching that affects optical schemes.

Employing two color pumps with integer frequency relations is required for establishing the symmetry-based photocurrent selection rules that we use for probing TRS breaking. This arises since noninteger frequencies (*e.g.,* ω-2.01ω) not only break TRS, but also lead to long driving periods where decoherence becomes relevant. Simple monochromatic drives are symmetric under inversion,^71^ just as any odd frequency ratio bichromatic field (e.g., 1:3, 3:5, etc.). Finally, for even/odd ratios, the appropriate φ_ω–2ω_ can be found where the bichromatic field exhibits TRS but lacks any other symmetry.

## Results

By measuring photocurrents along the *x*-direction as a function of the two degrees of freedom (θ_ω–2ω_ and φ_ω–2ω_), we now map out the symmetry-dependent photocurrent selection rules in graphene. Our main experimental data are presented in Figure 2, showing measured BPGE photocurrents. The currents exhibit a characteristic structure that reflects the combined symmetries of the material and optical electric field. To further understand these complex features, we analyze specific cases (Figure 2b­(i-iv)) for the θ_ω–2ω_-and φ_ω–2ω_-dependent photocurrent. Below, we show that all regions of Figure 2a exhibiting a photocurrent suppression result from either (or both) TRS and MS being maintained in the light-matter system.

**Figure 2:**
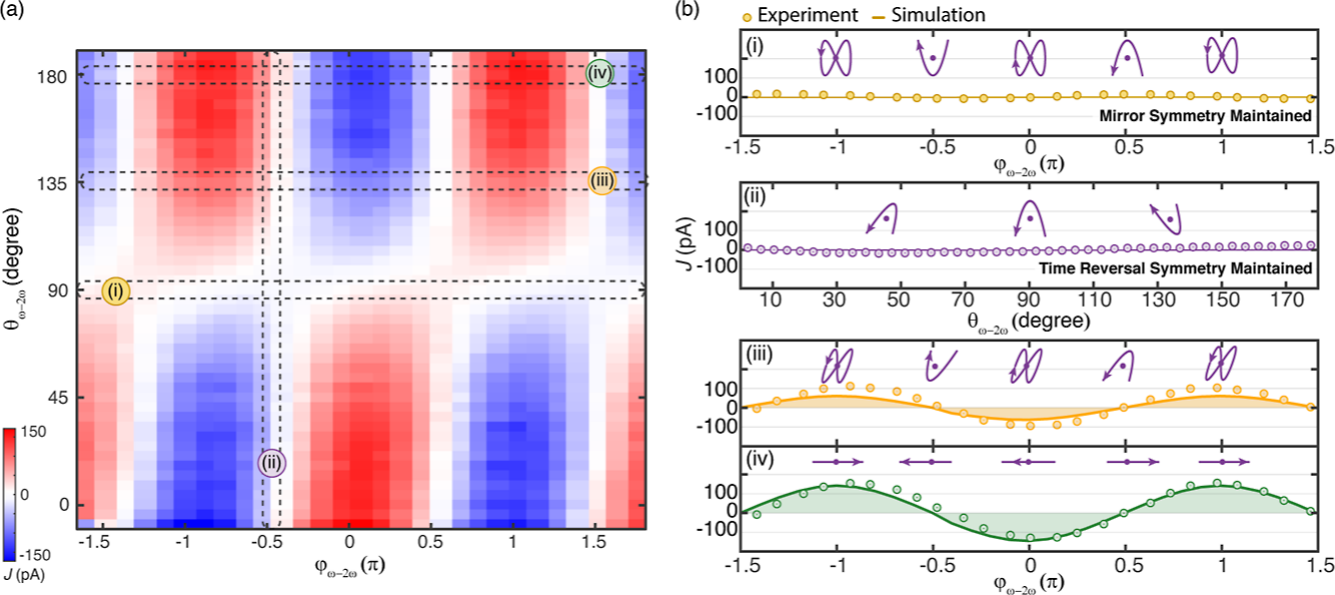
Two-color photocurrent spectroscopy. (a) Two-color phase (φ_ω–2ω_) and angle (θ_ω–2ω_)-dependent photocurrents in graphene. Regions of zero photocurrent correspond to waveforms that exhibit mirror and/or time-reversal symmetry. (b­(i-iv)) linecuts of (a) showcasing different symmetries. (b­(i)) Horizontal line-cut at θ_ω–2ω_ = 90° exhibits photocurrent suppression for all φ_ω–2ω_ due to the presence of mirror planes in the material. (b­(ii)) Vertical line-cut at φ_ω–2ω_ = π/2 showcasing photocurrent suppression for all θ_ω–2ω_ due to the material and waveform maintaining TRS for all phases. (b­(iii)) Linecut at θ_ω–2ω_ = 135°: both mirror and TRS are broken independently, allowing for φ_ω–2ω_-dependent photocurrent generation. (b­(iv)) Finally φ_ω–2ω_-dependent photocurrents, at θ_ω–2ω_ = 180°, are generated from the simultaneous and linked breaking of both mirror and TRS.

First, for θ_ω–2ω_ = 90° (Figure 2b­(i)), we observe a photocurrent suppression for all phases φ_ω–2ω_. Specifically, this example of a photocurrent suppression is a fundamental consequence of a more generalized dynamical mirror symmetry within the driving field, as discussed above, which was previously analyzed and observed in refs 25 and 76. This feature can be indicative of the presence of mirror planes in graphene, since if the selection rule is upheld, it follows that the material also exhibits the appropriate mirror plane. However, it is not helpful for deciphering if the system respects or breaks TRS.

Another robust feature in the photocurrent map (Figure 2a) is the minima for φ_ω–2ω_ = π/2 ± *m*π (for integer *m*) and all θ_ω–2ω_. Here, the tailored field is clearly polarized in the two-dimensional space of the monolayer plane (the *xy*-plane), for every choice of θ (Figure 2b­(ii)). In this case, mirror symmetry is broken by the optical field for any generic choice of θ_ω–2ω_ ≠ 0, 180° (see Lissajous figures inset in Figure 2b­(ii), implying that a different symmetry is imposing this selection rule. Indeed, it is apparent that these parameters invoke TRS for all θ_ω–2ω_ (i.e., **E**(*t*) = **E**(−*t*) regardless of the choice of θ_ω–2ω_). Therefore, the vanishing photocurrent is a result of a TRS-induced selection rule in the absence of any other symmetry relation that might cause such suppression. In that respect, by measuring photocurrents as a function of φ_ω–2ω_ across regions where the field’s symmetry is tuned, one can study the light-matter system as it transitions from a nonsymmetric to symmetric state via photocurrent spectroscopy. Vanishing photocurrents for the correct θ_ω–2ω_ and φ_ω–2ω_ (θ_ω–2ω_ ≠ 90°, φ_ω–2ω_ = π/2, for example, Figure 1c­(iii) indicate a symmetry-relation intrinsic to the material system, which can be probed by the photocurrent amplitudes. Further, the magnitude and location of the photocurrent nodes (i.e., their potential deviation from the expected selection rule) can be used as indicators for the extent of an intrinsically broken symmetry. Figure 2b­(iii,iv) show such a region where MS and TRS are broken and revived, allowing us to explore their individual roles in photocurrent generation. Finally, while the copolarized case (Figure 2b­(iv), explored extensively in the coherent control community^44–47^ ) also breaks MS and TRS, the light field still maintains a separate mirror element (since the field is polarized in 1D). This makes it ineffective to probe solely the TRS.

To further analyze these results, we performed ab initio simulations in similar conditions to the experiment, denoted by the solid lines in Figure 2b (see details in the Methods section). These agree remarkably well with our observations and corroborate the selection-rule origin of the effects (we rule out other potential contributions such as electronic correlations in ref 25).

Analytically, we can derive the consequences of TRS on induced BPGE currents by analyzing its impact on the excited carrier distribution throughout the Brillouin zone (BZ), generated by the bichromatic field. Ultimately, the symmetry of the conduction (and valence) band’s carrier *k*-space population determines whether a bulk photogalvanic current will be generated. In this case, we recall that *k* and –*k* are connected by TRS (with a complex conjugation operation connecting the Bloch states, ψ_k_ = ψ_‑k_^*^, where ψ_*k*_ is the Bloch state at *k*), meaning that band curvatures and velocities at *k* and –*k* are exactly inverted from one another for a given band. Moreover, because graphene is both inversion and time-reversal symmetric (zero Berry curvature throughout the BZ) any arising photocurrent can only be a result of electronic occupation in excited bands and their potential asymmetric configuration in the BZ (i.e., an injection current). However, a laser field preserving TRS, applied in a material preserving TRS, must excite an equal amount of carriers at *k* and –*k* for any point *k* in the BZ due to the degeneracy in the Bloch states being preserved by the laser driving. Critically, this means that TRS-preserving laser fields cannot generate BPGE photocurrents since the current generated by electrons occupying a given point in the BZ must be balanced by a reciprocal point. This selection rule is quite striking, especially considering the fact that the driving tailored field has a clear two-dimensional and spatially asymmetric configuration (see exemplary Lissajous figure in Figure 2b­(ii), which a priori appears as if it should excite a photocurrent. The photocurrent measurements presented in Figure 2a, specifically photocurrent suppression for all θ_ω–2ω_ of TRS-respecting waveforms in TRS-maintaining materials, represent the first experimental observation of the TRS-induced photocurrent selection rule by tailored light. Especially, it is the first observation of this selection rule in a light-matter system where other symmetries of the laser drive that could preclude photocurrents are absent (e.g., rotational or mirror planes that typically arise in monochromatic light).

Next, we show that the TRS-induced BPGE photocurrent suppression selection rule can be employed through symmetry-breaking spectroscopy to probe TRS-breaking phenomena. The logic of our proposal is as follows: Given an unknown sample where one would like to identify whether TRS is upheld (e.g., a ferromagnet), we should irradiate it with the tailored **E**_ω–2ω_(*t*) field while scanning θ_ω–2ω_ and φ_ω–2ω_. If no photocurrents are observed at the correct φ_ω–2ω_ for all θ_ω–2ω_ (φ_ω–2ω_ = π/2), TRS is preserved. Otherwise, it is broken, and the extent to which it is broken should correlate with the degree of intrinsic TRS breaking in the sample. Remarkably, this approach does not require actually breaking TRS in the probe pulse, as is commonly done either in circular dichroism measurements or by applying external magnetic fields: the critical information is embedded at the parameter point where the two-color driving field respects TRS. The approach is also inherently ultrafast time-resolved, enabled by the optical pulses, which would be challenging to achieve with external magnetic fields. This generates a background-free signal for the symmetry-broken phases and can be easily multiplexed with other measurement techniques.

We tested this concept in ab initio simulations (see Figure 3). First, we turn our attention to a two-dimensional magnet, CrI_3_.^67^ From a material point of view, CrI_3_ comprises a 2D hexagonal lattice similar to graphene, with the lattice structure and electronic band structure presented in Figure 3a (see Methods for technical details). Importantly, it is ferromagnetic in its ground state with nonzero magnetic moments on Cr *d*-states, which inherently breaks TRS. From a light-matter interaction perspective, these magnetic moments should cause deviations from the expected photocurrent suppression selection rule due to the broken TRS. Indeed, this deviation is present in the φ_ω–2ω_-dependent photocurrents: In Figure 3b, we compare the φ_ω–2ω_-dependent photocurrent generation in graphene (yellow, experimental and ab initio) to the predicted photocurrents from CrI_3_ (blue, ab initio), for a θ_ω–2ω_ = 135°. The small percent level difference between φ_ω–2ω_ = 0 and φ_ω–2ω_ = π is most likely due to the finite pulse duration of the experimental pulses as well as the simulated fields. Importantly, we observe a strong deviation from the typical curve observed in graphene: The minimal photocurrent response is predicted at φ_ω–2ω_ = 0 instead of φ_ω–2ω_ = π/2, and it never reaches a zero value.

**Figure 3:**
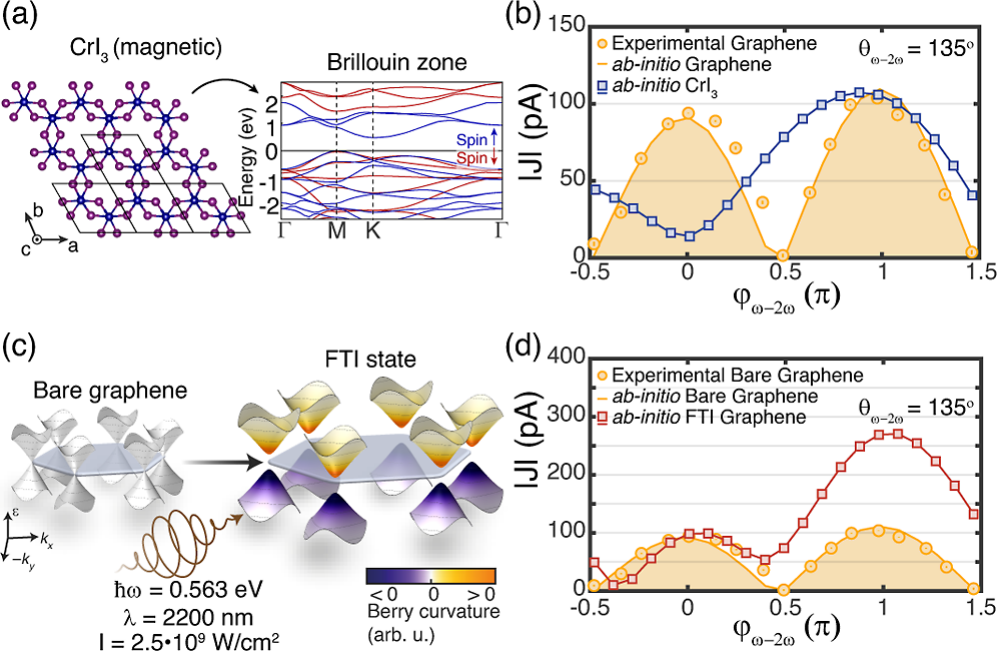
Extension to time-reversal symmetry-broken systems. (a) Real space depiction of the two-dimensional ferromagnet CrI_3_ and its Brillouin zone. Blue and purple atoms represent chromium and iodine, respectively, while the blue and red bands in momentum space are for spin up/down, respectively. (b) ab initio TDDFT simulation of φ_ω–2ω_-dependent photocurrents in CrI_3_ at θ_ω–2ω_ = 135° overlaid with both measured and simulated graphene photocurrents at identical laser parameters. See text for discussion. (c) Momentum space depiction of optically dressed graphene with a circular field at 2200 nm, resulting in an FTI state (with a 100 meV bandgap) with broken time-reversal symmetry, which we probe by tailored light photocurrent spectroscopy. (d) Ab initio TDDFT simulation of φ_ω–2ω_-dependent photocurrents in the FTI state at θ_ω–2ω_ = 135° overlaid with both measured and simulated graphene photocurrents at identical laser parameters. Clearly, we can detect the TRS breaking of the FTI state.

Next, we investigate a TRS-broken system resulting from optical dressing, Figure 3c. By irradiating (i.e., dressing) the graphene sample with a circularly polarized mid-infrared pulse (at 2200 nm), we cause it to enter into a Floquet Chern insulating state,^68,69^ opening a topological gap. Notably, due to the dressing field’s rotational symmetry, it cannot induce a photocurrent in graphene on its own. With these dressing conditions, the fundamental topological gap opening from the circular beam is small but observable (∼0.1 eV for the highest field strength). Due to the dressing frequency, intensity, and graphene band structure, multiple topological band gaps open at avoided crossings of the Floquet replica. Each band gap can exhibit Berry curvature, contributing to the overall topological state.^77^ Importantly, the ω – 2ω probe field in the symmetric configuration (Figure 1c­(iii), θ_ω–2ω_ = 135°, φ_ω–2ω_ = π/2) preserves TRS and therefore cannot open a topological gap itself,^40^ regardless of the complexity of the Floquet bands. By exploring the photocurrent response with respect to the ω – 2ω relative phase (at θ_ω–2ω_ = 135°, Figure 3d), we observe a distinct deviation from the typical selection rule: Nonvanishing photocurrents arise for any choice of φ_ω–2ω_, starkly different than the bare graphene case, both from the ab initio simulation and experimentally measured. The impact of TRS breaking from the dressing field is directly evident when observing the nonzero photocurrent at φ_ω–2ω_ = π/2 as well as the increased asymmetry of the φ_ω–2ω_-dependent photocurrent. Furthermore, the minimum φ_ω–2ω_-dependent photocurrent is slightly shifted to φ_ω–2ω_ = 0.45π, reflecting that TRS is only marginally broken in the Floquet Chern insulator. Nevertheless, this small shift proves that symmetry-breaking spectroscopy can be applied to probe-driven quantum phases of matter. Such a multidimensional spectroscopy scheme might also encode information about the Floquet bands’ Berry curvature distribution as well as the gap size,^78^ which should be imprinted onto the photocurrent but is beyond our current scope.

We further explore the φ_ω–2ω_-dependent photocurrent generation in an FTI by varying the peak intensity of the 2200 nm dressing pulse. As the intensity of the dressing field increases, two effects are apparent. First, the gap size at K/K′ increases with the applied dressing field.^68,69^ Second, the Berry curvature of the bands spreads from K/K′ as the dressing field increases, as shown schematically in Figure 4a. Through ab initio simulations, we predict φ_ω–2ω_-dependent photocurrents for θ_ω–2ω_ = 135° as a function of the dressing intensity (Figure 4b). Three key features emerge. The first is that the photocurrent magnitude increases with increasing dressing intensity, as intuitively expected for more strongly driven systems. The second is that the minimum photocurrent position shifts away from the time-reversal symmetric waveform minimum of φ_ω–2ω_ = π/2. Finally, qualitatively, the minimum photocurrent (at φ_ω–2ω_ = – 0.45π) is nonzero for low dressing intensities, decreases as the dressing field increases, and increases again as the dressing field reaches its maximum. This could be thought of as a measure of the degree of time-reversal symmetry breaking of the FTI state. When the FTI’s state weakly breaks time-reversal symmetry, a bichromatic field with the opposite chirality can effectively restore the symmetry of the system (albeit at a different φ_ω–2ω_ than the TRS-maintaining system). While the total combined (dressing and two-color probe) waveform is exceedingly complex, this φ_ω–2ω_-dependent photocurrent could realize a way to probe the magnitude and sign of the degree of time-reversal symmetry breaking in light-dressed states.

**Figure 4:**
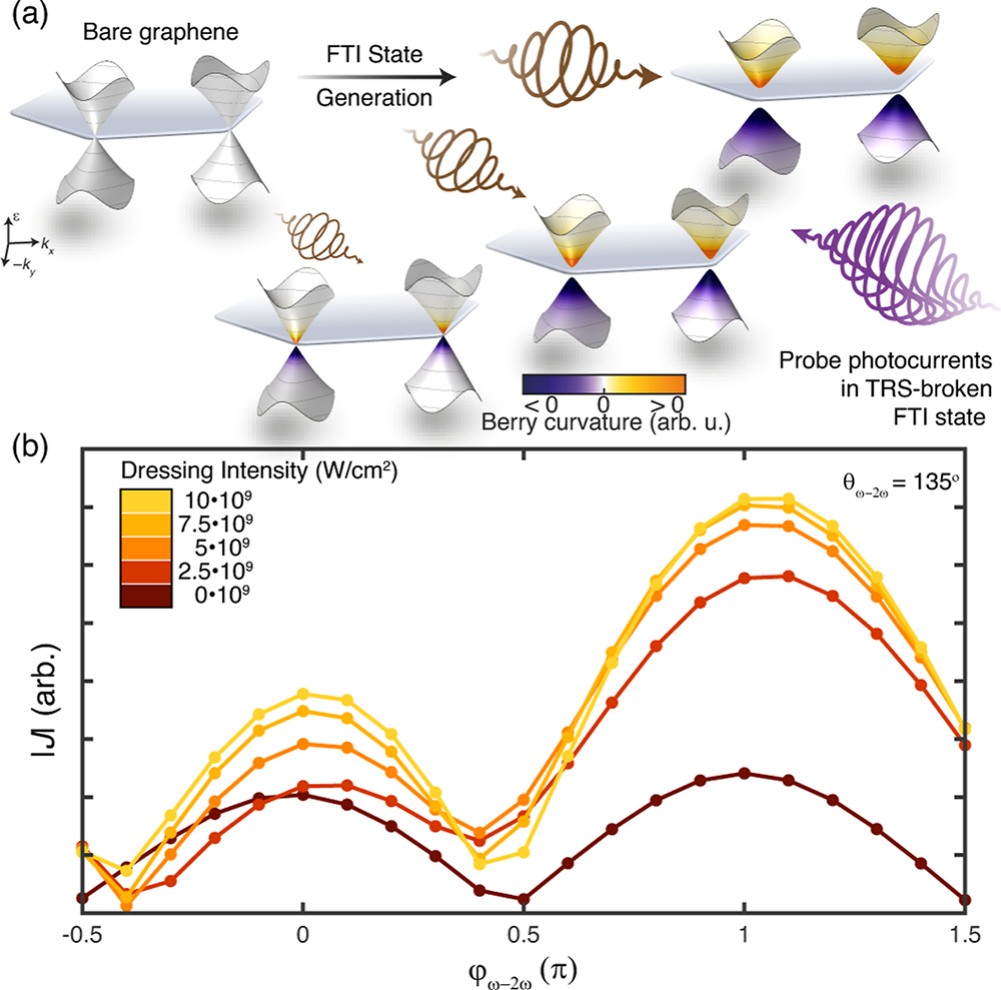
Probing time-reversal symmetry in an FTI. (a) Schematically, bare graphene is illuminated with a circular field with a central wavelength of 2200 nm. As the dressing field increases in intensity, the band gap increases and the Berry curvature spreads from the *K*/*K*′ points (seen illustratively by the colors of the bands and the band gap). (b) Simulated ab initio two-color photocurrents from dressed graphene, at a variety of dressing intensities (color bar for scale). Three key observations can be made. (i) Trivially, as the dressing field increases, more photocurrent is generated. (ii) Due to the broken time-reversal symmetry, the current minimum at φ_ω–2ω_ = 0.5π shifts to φ_ω–2ω_ = 0.45π. (iii) Finally, the degree of time-reversal breaking can be seen from the decrease (and then reversal) of the dressing field intensity-dependent photocurrent at φ_ω–2ω_ = – 0.45π. We observe this behavior because the degree of time-reversal symmetry breaking for the photocurrent-generating waveform is similar in magnitude but opposite in sign to the degree of time-reversal symmetry breaking of the FTI state.

Our simulations, complemented by experimental observations in graphene, confirm the ability to probe TRS-broken phases using tailored TRS-respecting light probes (absent of circularly polarized components). Most importantly, the signal is effectively background-free and potentially ultrafast time-resolved. This approach should open up new opportunities for exploring ultrafast magnetism,^57^ steady-state Floquet phases driven by monochromatic^24,79^ or tailored light,^71^ and phonon-driven magnetism,^80,81^ as well as other magnetic mechanisms,^82^ and finally chirality-induced-spin-selectivity.^83^

## Discussion and Outlook

To summarize, we experimentally and theoretically explored tailored light-driven bulk photogalvanic currents in graphene and predicted their response in other hexagonal 2D systems. We showed that for a unique case, where the tailored field comprises bichromatic linearly polarized ω – 2ω components, one can engineer time-reversal symmetry and mirror symmetry-respecting/breaking laser pulses by scanning both the polarization angle (θ_ω–2ω_) and two-color phase (φ_ω–2ω_). This showcases the bichromatic field’s ability to probe phases of matter without explicitly breaking their symmetry, as commonly done with monochromatic fields. We measured this tailored-light photocurrent response in graphene with respect to θ_ω–2ω_ and φ_ω–2ω_, allowing us to demonstrate TRS-induced photocurrent suppression selection rules. This is the first observation of such selection rules and is unique in the ability to isolate the effect of TRS from other symmetries in the laser field. We analyzed this result both analytically and with state-of-the-art ab initio simulations, corroborating the interpretation and physical mechanism. Utilizing tailored light photocurrent spectroscopy, we performed calculations in more complex quantum systems, showing that the TRS-induced selection rule is broken in phases of matter with broken TRS such as a 2D magnet and a Floquet Chern insulator (whose degree of TRS-breaking correlates with the topological gap size and Berry curvature distribution). Furthermore, our tailored-light photocurrent spectroscopic concept can be multiplexed with other forms of optical spectroscopy using the same source to achieve multidimensional information. Finally, our approach can be applied to unknown material symmetries to elucidate the material symmetries. Our work predicts clear signatures of TRS-broken phases in tailored light photocurrent spectroscopy and permits probing such phases without circularly polarized fields and/or external magnetic fields, which can themselves alter the state being explored. While we have explored ferromagnetic and topological systems with broken TRS, an interesting future candidate would be altermagnets,^84^ with intrinsic symmetries that might make photocurrent signals even more intricate. Our symmetry-based ultrafast technique should provide an experimentally accessible and useful spectroscopic tool for ultrafast magnetism^57–59^ and phase transitions.

Looking forward, these results should also impact other ultrafast spectroscopies of various physical and chemical systems such as chirality^30^ and chirality-induced spin selectivity,^85^ where novel methods are needed. We believe that these results could bridge the fields of highly nonlinear optics in solids, exploring tailored light responses such as high harmonic generation,^34,73^ and nonlinear photocurrent excitation,^21^ allowing the two effects to be analyzed on an equal footing.^40,76^

## Methods

### Experimental Methods

For producing the biharmonic tailored fields, we frequency double an Erbium fiber frequency comb^86^ at 80 MHz in a 1 mm BiBO crystal, producing 213 and 110 fs pulses for the fundamental (1550 nm) and second harmonic (775 nm), respectively, identical to ref 25 . The delay between the two fields (φ_ω–2ω_) is controlled by a calcite inline interferometer,^70^ while the angle of the second harmonic is controlled by a two-color half-wave plate (Newlight Photonics, θ_ω–2ω_), shown schematically in Figure 1b. The biharmonic tailored fields are then focused with an off-axis parabolic mirror to 0.27 and 0.2 V/nm for the fundamental and second harmonic, respectively, on monolayer epitaxially grown graphene on SiC. The graphene on SiC is placed within a vacuum chamber at 10^–8^ hPa and at room temperature. Finally, φ_ω–2ω_-dependent photocurrents (green arrow) are measured using gold electrodes in the *x*-direction using a lock-in amplifier referenced to the φ_ω–2ω_. Further experimental details are provided in ref 25.

### TDDFT Simulations

This section describes technical details of the ab initio simulations presented in the main text. All calculations were performed with the open access code Octopus.^87^ The ground states of graphene and CrI_3_ were obtained at the hexagonally symmetric lattice with lattice parameters 4.65 and 7 Bohr, respectively, where otherwise symmetries over Bloch states were not imposed. Γ-Centered k-grids of sizes 120 × 120 and 15 × 15 were employed in graphene and CrI_3_, respectively. We employed grid spacings of 0.38 and 0.39 Bohr and a *z*-axis of sizes 60 and 50 Bohr for graphene and CrI_3_, respectively. Time-dependent simulations were initiated from the ground-state electron configuration at *t* = 0. For CrI_3_, we employed spin-DFT, leading to a magnetic ground state with a magnetization of 3.2 Bohr on each Cr atom and a semiconducting phase.

We solved the time-dependent Kohn–Sham (KS) equations of motion on a real-space grid in the length gauge

1 $$i\partial_{t}\psi_{n,k}\left(r,t\right)=\left(\frac{1}{2}{\left(-i\nabla +A\left(t\right)/c\right)}^{2}+v_{KS}\left(r,t\right)\right)\psi_{n,k}\left(r,t\right)$$

where ψ_*n*,*k*_(*r*, *t*) is the KS state at band *n* and *k*-point *k*, and *v*_KS_ is the KS potential comprising a classical Hartree term, the interactions of electrons with nuclei and deeper core states (incorporating norm-conserving pseudopotentials^88^), and the exchange–correlation (XC) potential. We employed the adiabatic local density approximation (aLDA) for the XC functional throughout. Periodic boundaries were employed in the monolayer plane, while the *z*-axis was treated with finite boundary conditions. A complex absorber of width 12 Bohr was added during propagation (similar to the approach in ref 76). In eq 1, **A**(*t*) is the applied vector potential within the dipole approximation

2 $$A\left(t\right)=f\left(t\right)\frac{cE_{0}}{\omega }\left(\cos\left(\omega t+\varphi_{\omega -2\omega }\right)ŷ+\frac{\Delta }{2}\left(\cos\left(\theta \right)\cos\left(2\omega t\right)\mathrm{x̂}+\sin\left(\theta \right)\sin\left(2\omega t\right)ŷ\right)\right)$$

where *c* is the speed of light, *E*_0_ is the electric field amplitude (taken at the experimental values), Δ = 0.75 is the field amplitude ratio, φ_ω–2ω_ the two-color phase, ϵ the ellipticity of the 2ω field, ω the carrier frequency (taken at the experimental value corresponding to 1550 nm light), and *f*(*t*) a temporal envelope taken as a ’supersine’ form^89^

3 $$f\left(t\right)=\sin{\left(\pi \frac{t}{T_{p}}\right)}^{\left(\left|\pi \left(\frac{t}{T_{p}}-\frac{1}{2}\right)\right|/\sigma \right)}$$

where σ = 0.75, *T*_p_ is the laser pulse duration chosen as *T*_p_ = 8*T*, and *T* = 5.17 fs is a single cycle of the ω carrier frequency. The angle θ is the relative angle between the two carrier wave polarizations, which are taken to be linearly polarized at all configurations.

The KS equations were propagated in the absence of scattering and decoherence, from which we obtained the time-dependent KS states. The total time-dependent light-driven photocurrent was calculated by

4 $$J\left(t\right)=\sum_{n,k}w_{k}\int \left[\psi_{n,k}^{*}\left(r,t\right)\left(\frac{1}{2}\left(-i\nabla +A\left(t\right)/c\right)-i\left[V_{ion},r\right]\right)\psi_{n,k}\left(r,t\right)\right]dr+c.c.$$

where *V*_ion_ is the nonlocal part of *v*_KS_ (due to the pseudopotentials), *w*_*k*_ is the *k*-point weight, and the sum is performed over occupied KS states. From **J**(*t*) the injection current was evaluated by averaging over a single cycle of the fundamental frequency after the driving laser pulse has ended (at *t* = *t*_*f*_): $j_{photo}=\int_{t_{f}}^{t_{f}+T}J\left(t\right)dt$ .

In the case of the Chern insulating phase, the same procedures were employed as in the bare graphene simulations, except that an additional laser pulse centered at 2200 nm with a circular polarization of the *xy*-plane was incorporated, with the same supersine temporal envelope.

Since the simulations are performed in the velocity gauge, the current contributions from intraband and interband dynamics cannot be resolved within this scheme but could carry further information on electron dynamics.

## Supplementary Material



## Notes

Optical field strength calculation, photocurrent scan for the TRS preserving case, and photocurrent numerical convergence (PDF)
