# Supplementary material for: Probing Broken Time-Reversal Symmetry in 2D Materials with Tailored-Light Photocurrent Generation
Source: ACS Nano. 2026 Mar 17;20(15):11614–23. doi: 10.1021/acsnano.5c17857 (PMC13334570; doi:10.1021/acsnano.5c17857)
Supplement: Supplementary file 1 [file nn5c17857_si_001.pdf]

Supplementary Info: Probing broken time-reversal symmetry in 2D materials with tailored-light photocurrent generation

Daniel M. B. Lesko,[*,†,‡] Tobias Weitz,[†,‡] Simon Wittigschlager,[†] Selina Nöcker,[†,‡] Weizhe Li,[†] Peter Hommelhoff,[*,†,‡] and Ofer Neufeld[*,¶]

†*Department of Physics, Friedrich-Alexander-Universität Erlangen-Nürnberg (FAU), Erlangen, 91058, Germany*

‡*Fakultät für Physik, Ludwig-Maximilians-Universität, München, 80539, Germany*

¶*Technion Israel Institute of Technology, Faculty of Chemistry, Haifa 3200003, Israel*

E-mail: Daniel.Lesko@fau.de; Peter.Hommelhoff@fau.de; Ofern@technion.ac.il

Supplemental Information

Field strength calculation

To calculate the peak electric field strength for a given intensity, the following equation can be used,

$$E_0 = \sqrt{\frac{2 \times I_0}{c \times \epsilon_0}}, \tag{S1}$$

where I_0 is the intensity, c is the vacuum speed of light, and ϵ_0 is the vacuum permittivity.

Time reversal symmetry preserving scan

To showcase that the photocurrent suppression comes from the material's TRS, and not an accidental symmetry, we show ab-initio photocurrent generation simulations from both graphene and CrI_3 for the case of a TRS-waveform as above for varying relative angles ($\varphi_{\omega-2\omega} = 0.5\pi$, Fig. S1). We see large photocurrent signals for all waveforms that exhibit TRS in CrI_3, and photocurrent suppression in graphene. Notably, the photocurrent signal in CrI_3 in this scan is very weakly-dependent on the relative angle, which is in agreement with our interpretation connecting it the the degree of TRS breaking - in this case the field perfectly respects TRS and any broken TRS is intrinsic to the magnet.

Numerical convergence

Figure S2 shows convergence of photocurrent signals vs k-grid density in both material systems explored here. Note that for graphene convergence is tested at central wavelengths of 2500 nm for slightly different two-color field parameters than those employed in the main text. Time-steps and real-space grids were similary converged (not shown).

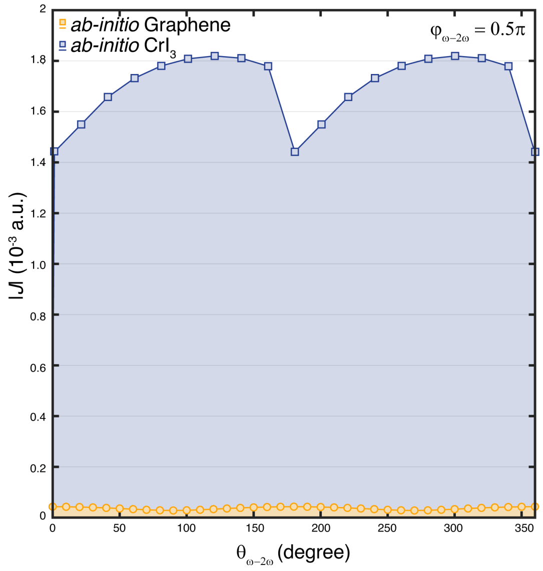

Figure S1: **Time-reversal symmetric waveform scan.** Ab-initio two-color photocurrents from graphene and CrI_3 as a function of $\theta_{\omega-2\omega}$ for a $\varphi_{\omega-2\omega} = 0.5\pi$. Graphene shows a strong photocurrent suppression as the material and two-color field both maintain time-reversal symmetry for all $\theta_{\omega-2\omega}$. CrI_3 shows strong photocurrent generation due to its ferromagnetic nature, despite the two-color waveform maintaining TRS.

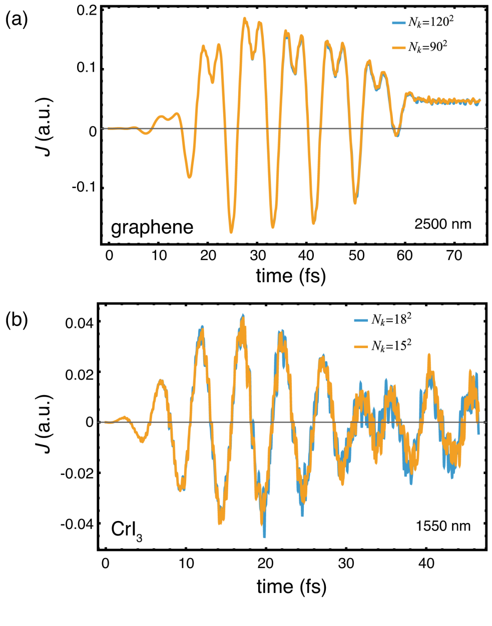

Figure S2: **Numerical convergence vs k-grid density.** Ab-initio two-color photocurrent evolution from graphene, **(a)**, and CrI_3, **(b)**.
